# Do Women with Diabetes Need More Intensive Action for Cardiovascular Reduction than Men with Diabetes?

**DOI:** 10.1007/s11892-020-01348-2

**Published:** 2020-10-09

**Authors:** Jürgen Harreiter, Helena Fadl, Alexandra Kautzky-Willer, David Simmons

**Affiliations:** 1 Gender Medicine Unit, Division of Endocrinology and Metabolism, Department of Medicine III, Medical University of Vienna, Spitalgasse 23, 1090 Vienna, Austria; 2 Department of Obstetrics and Gynaecology, Faculty of Medicine and Health, Örebro University, SE 70182 Örebro, Sweden; 3 Gender Institute, Gars am Kamp, Austria; 4 Macarthur Clinical School, Western Sydney University, Sydney, New South Wales Australia

**Keywords:** Diabetes mellitus, Gestational diabetes, Cardiovascular disease, Sex, Gender, Prevention, Diabetes management, CVD risk factors, Lipids, Sex hormones

## Abstract

**Purpose of Review:**

This narrative review makes the case for greater efforts to reduce cardiovascular disease (CVD) risk in women with diabetes.

**Recent Findings:**

In a recent meta-analysis including five CVOTs of diabetes medications with 46,606 subjects, women (vs men) with type 2 diabetes had a higher relative risk for stroke (RR 1.28; 95% CI 1.09, 1.50) and heart failure (1.30; 1.21, 1.40). Prior studies found higher “within-gender” RR for CVD mortality in women with diabetes although men have an absolute higher risk. Women with prior gestational diabetes mellitus (GDM) have a 2-fold higher CVD risk than the background population. Worse CVD and CVD risk factor management in women, as well as lower female therapy adherence, contribute further to these disparities.

**Summary:**

The mechanism behind this excess risk includes biological, hormonal, socioeconomic, clinical, and behavioral factors that still require further investigation. The need for more intensive CVD reduction in women now includes more attention to screening for both incident diabetes and CVD risk factors among high-risk women.

## Introduction

In the general population, women die at an older age than men in nearly all parts of the world such that life expectancy is on average 4 years higher among women than men [1]. These differences have been attributed to biological, socioeconomic, environmental, and behavioral factors [2–4]. Although women live longer, this advantage frequently fails to translate into increased healthy life years [5]. Cardiovascular disease (CVD) is the leading cause of death among both men and women [6]. Diabetes is a well-known CVD risk factor and thus patients with diabetes mellitus have a high need for approaches that will reduce premature mortality from CVD. The number of people with diabetes worldwide is predicted to increase from the current 463 million to 700 million by 2045 [7], and with it, the number of people developing CVD associated with diabetes. While there are only small gender differences in the age-specific prevalence of diabetes in adulthood [8••], there are sex and gender differences in the relationship between diabetes and CVD mortality.

Since the Framingham study is nearly five decades ago, it has been clear that the usual “female protection” is lost among women with insulin-treated diabetes, and that this is based on their higher CVD risk [9]. Several other studies subsequently demonstrated the increased relative risk for CVD in women with diabetes compared with men, in both type 1 (T1DM) and type 2 (T2DM) diabetes [10•, 11, 12•, 13••]. An excess CVD risk has also been shown among women with prior gestational diabetes mellitus (GDM) [14••]. The exact pathophysiological explanations remain under-investigated and need further research, but the higher risk seems to be driven by biological, environmental, and behavioral factors [8••]. Biological causes discussed include a loss of the protective hormonal effect by female sex hormones and sex hormone imbalance in hyperglycemic conditions leading to higher oxidative stress and endothelial dysfunction, a proinflammatory environment acting on estrogen receptor actions, a modulation of vascular response to nitric oxide, and impaired vessel relaxation properties [8••]. Further proposed explanations for the excess CVD mortality reported in women with diabetes compared with those without diabetes include pregnancy-related conditions and sex and gender differences in diabetes management and the management of other CVD risk factors [15] including hypertension [16].

In view of this sex and gender difference in excess CVD risk, our narrative review poses the question, whether all or, at least some women with diabetes, are in need of increased and more aggressive cardiovascular risk reduction than men. This includes the need for any further advances in diabetes prevention, treatment, CVD risk reduction, or management. We further attempt to shed light on the biological and socio-cultural aspects that are potentially responsible for these sex- and gender-related disparities.

## Why Do Women Live longer?

In general, in high-income populations, life expectancy is 4–7 years higher in women than in men [17], whereas in low-income countries, a smaller gap was reported as women have less access to health care services [1]. Between the poorest and richest countries, the life expectancy gap opens up to more than 18 years as reported in a 2018 WHO report [1]. There are some data to suggest that this survival difference is mainly due to modifiable risk factors with biological causes only playing a minor role. Key evidence comes from a study comparing 11,000 German male and female cloister inhabitants from Catholic communities with the general population. A maximum survival disadvantage of about 1 year of life expectancy from a young adult age (25 years) was found in the male cloister inhabitants compared with women from the cloister communities and the general population [18]. Men from the general population had a lower life expectancy from a young adult age compared with the cloister inhabitants. It was also proposed that these males were unable to decrease their mortality risk, in contrast to the female general population, nuns, and monks [18]. The protected environment of the cloister seemed to have positive effects on life expectancy on male inhabitants. Modifiable risk factors, such as smoking, alcohol, unhealthy food choices, excess body weight, and lack of physical inactivity, were more prevalent in the male general population [18].

While women have longer life expectancy, there is evidence to suggest that the balance between healthy and unhealthy live years includes a sex-specific imbalance, the so-called health survival paradox [5]. While women and men spend comparable time in a “good health” state, the longer life expectancy in women results in women accumulating more unhealthy life years compared with men.

## Increased Mortality Risk in Women with Diabetes?

In general, the absolute risk for T2DM and CVD mortality is higher in men; however, the relative risk of all-cause and CVD mortality in women with T2DM has consistently been reported to be significantly higher and was also reported in women with T1DM (Table 1) [12•, 19, 20, 21•, 22, 23]. Higher risks were observed in younger groups with diabetes compared with older groups with the highest risk found in women with diabetes between 35 and 59 years of age [22]. Some studies observed higher CVD risk in both sexes, with a non-significant higher relative risk in women [23]. Key findings pertinent to sex differences in CVD outcomes were the reported higher obesity, hypertension, and dyslipidemia, as well as lower prescription of medication among women [23]. Furthermore, the risk of heart failure was reportedly higher in women with T1DM and T2DM (Table 1) [24•, 25•]. There was a narrowing sex ratio within older age categories, indicating that relative risk is higher in women of younger age [24•].

**Table 1:** Studies reporting sex and gender differences in the effect of diabetes mellitus on all cause and cardiovascular disease mortality and heart failure

Author	Population	Design	Outcome
Cardiovascular mortality			
Xu et al. [19]	2,314,292 individuals, among whom 254,038 all-cause deaths occurred	Systematic review and meta-analysis including prospective cohort 35 studies	Pooled women vs. men ratio of the HRs 1.17 (95% CI: 1.12–1.23) and 1.97 (1.49–2.61) respectively for all cause and coronary heart disease (CHD) mortality. increased all-cause mortality in men (HR 1.91 (1.72–2.12)) and women (2.33 (2.02–2.69)) with T2DM vs. healthy population
Wang et al. [20]	5,162,654 participants	Systematic review and meta-analysis including 49 studies	Higher relative all-cause mortality (RRR 1.13, 95% CI 1.07–1.19; *p* < 0.001) and CVD mortality (1.30, 1.13–1.49; *p* < 0.001) in women with diabetes vs. men with diabetes. Women with diabetes vs. men with diabetes: CHD mortality RRR 1.58, 95% CI 1.32–1.90; *p* < 0.001 and stroke mortality 1.08, 1.01–1.15; *p* < 0.001
Huxley et al. [21•]	447,064 participants	Meta-analysis of 37 prospective cohort studies	Higher risk of fatal CHD in women with diabetes vs. men, pooled ratio of the RR 1.46 (1.14–1.88). Fatal CHD in patients with diabetes vs. no diabetes significantly higher in women than men: 3.50 (2.70–4.53) vs. 2.06 (1.81–2.34).
Huxley et al. [12•]	214,000 participants and 15,273 events	Meta-analysis of 26 studies	pooled women-to-men ratio in patients with T1DM: SMR 1.37 (95% CI 1.21–1.56) for all-cause mortality, 1.44 (1.02–2.05) increased risk for renal disease mortality, 1.37 (1.03–1.81) for stroke mortality, CVD mortality 1.86 (1.62–2.15), 2.54 (1.80–3.60) for incident coronary heart disease
Prospective Studies Collaboration and Asia Pacific Cohort Studies Collaboration [22]	980,793 participants and 76,965 fatalities different ethnicities and age categories, age 35–89 years	Meta-analysis of 68 prospective studies	Doubling of occlusive vascular mortality risk in men with diabetes RR 2.10, 95% CI 1.97–2.24), tripling in women with diabetes (3.00, 2.71–3.33) after stratification for age, total cholesterol, blood pressure, smoking status and BMI. Higher risks in younger groups with diabetes aged 35–59 years (2.60, 2.30–2.94) vs. older groups aged 70–89 years (2.01, 1.85–2.19), highest risk in women with diabetes and age 35 and 59 years (5.55, 4.15–7.44). in absolute numbers, adjusted diabetes associated excess occlusive vascular mortality comparable in men and women across all age categories
Wright et al. [23]	79,985 patients with incident T2DM between 2006 and 2013 matched to 386,547 patients without T2DM	Retrospective cohort study	Higher CVD event risk in both men and women, with a non-significant higher relative risk in women (RR 1.07 (0.98–1.17)
Clemens et al. [13••]	46,606 participants of trials examining the effect of diabetes medications on major adverse cardiovascular events in people ≥18 years of age with T2DM	Meta-analysis of 5 CVOTs on 3- or 4-point MACE (i.e., CV death, nonfatal myocardial infarction, nonfatal stroke, hospitalization for heart failure, hospitalization for unstable angina for 4-point MACE)	Higher risk for women for stroke (RR 1.28; 1.09–1.50), heart failure (1.30; 1.21–1.40), and CKD (1.33; 1.17–1.51), similar risk for PAD (1.12; 0.97–1.30) and lower risk for myocardial infarction (0.71; 0.59–0.86), consistently fewer female participants (28.5–35.8%) in the trials.
Heart failure			
Malmborg et al. [24•]	218,549 (46% women) participants, age 40–89 years	Population based study	Higher absolute risk of MACE-HF in men, but in relative terms 15% higher women to men ratio (95% Cl 1.11–1.19, *p* < 0.001) at the age of 50–60 years. The risk of recurrent CVD events or HF within 30 days irrespective of diabetes or sex, more often with increasing age.
Ohkuma et al. [25•].	12,142,998 individuals and 253,260 heart failure events	Systematic review and meta-analysis of 47 cohorts	Women with diabetes have increased relative risk of heart failure vs. men: T1DM (RRR 1.47 (1.44, 1.90)), T2DM (RRR 1.09 (1.05, 1.13))
Kannel et al. [26].	5209 men and women, age 30–62 years,	Framingham study, 18 year follow up	Five- and two-fold increased risk of heart failure in women and men with diabetes respectively vs. healthy population

*HR*, hazard ratios; *RRR*, relative risk reduction, defined as the relative decrease in the risk of an event in a cohort exposed compared with a cohort unexposed to a disease/risk; *SMR*, standardized mortality ratio, defined as an age and sex matched comparison of mortality in a cohort with a specific illness to controls; *RR*, relative risk, defined as the ratio of the likelihood of an event in an exposed compared to an unexposed cohort; *CVOT*, cardiovascular outcome trial

**Fig. 1:**
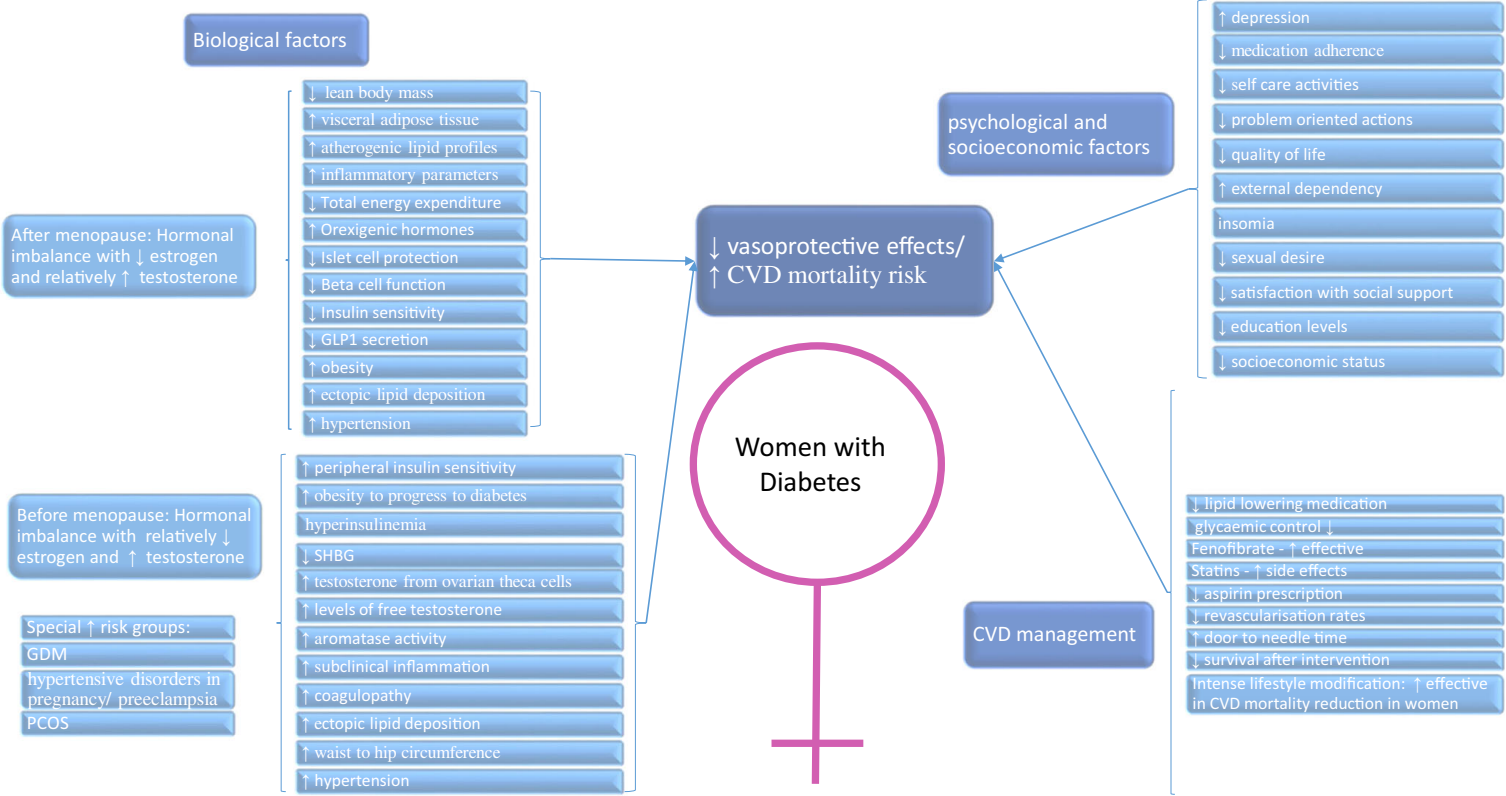
Specific risk factors for progression to CVD and CVD mortality in women with diabetes mellitus

Other studies also hypothesize a reduction in sex-related protection in women with diabetes, which is, in turn, associated with higher mortality risk [8••, 27, 28]. Potential risk factors explaining these differences are shown in Fig. 1. In view of this substantial effect of diabetes on CVD risk, there have been discussions over the need for more intensive CVD reduction activities, and individualization of risk reduction approaches [8••, 29, 30•]. It is obvious from the presented data that an individualized risk reduction approach needs to take into account both sex and age (e.g., the use of CVD risk reduction agents in women of reproductive age), but there also needs to be a focus on modifiable risk factors.

## Are the Differences in Excess CVD Mortality Due to Sex Hormone Differences?

Changes in hormonal action might contribute to the faster progression of CVD in both men and women with diabetes as a result of the effects on CVD risk factors as insulin resistance, overweight and obesity, and subsequently hyperglycemia, hypertension, and hypercholesterolemia evolve [31•]. The loss of female protection is therefore potentially driven by hormonal imbalance with the increased atherogenic lipid profiles, higher inflammation, or stronger relation of other CVD risk factors with hyperglycemia in women than men [8••, 28]. The contribution to the excess CVD among women with diabetes by pregnancy complications such as gestational hypertension and preeclampsia are also not fully understood, but early-onset preeclampsia has been shown to be associated with risk factors for CVD by the fifth decade of life [32].

Menopause is a particularly important time for CVD risk factors, when female sex hormone levels (e.g., estrogen) suddenly drop associated with a relative increase in male hormones (e.g., testosterone). This change is associated with weight gain, impairments in glucose metabolism and lipid storage, and finally progression to T2DM [8••]. After menopause, lean body mass declines and the prevalence of obesity increases [33••, 34]. The low estrogen levels after menopause reduce total energy expenditure through effects on the hypothalamus and higher release of orexigenic hormones [34]. Higher estrogen levels are also related to increased beta cell function and improved glycemic control in women with T2DM, and, additionally, slightly lower T2DM risk was reported in studies administering hormone replacement therapy in post-/menopausal healthy women [35••]. Between the ages of 50 and 59 years, and within 10 years after menopause onset, hormone replacement therapy is effective in preventing the development of T2DM and cardiovascular disease in women without diabetes, although safety concerns persist with the need to balance benefits with risks [35••]. Endogenous estrogen in women might exert vasoprotective effects and thus lower cardiovascular events in women—a benefit which seems attenuated in women with hyperglycemia [8••, 31•].

Female sex hormones and especially endogenous estrogen seem to have beneficial and protective effects on islet cells by preventing islet cell apoptosis driven by oxidative stress and lipotoxicity [36]. Higher insulin production and secretion were found along with approximately 6% higher beta cell content in pancreatic biopsies of women compared with men. Higher insulin secretion in women was associated with sex differences in glucagon-like peptide-1 (GLP-1) secretion, which was found to be 20% higher in normo-glucose-tolerant women compared with healthy men [36]. These differences found between women and men did not exist after progression to prediabetes or diabetes, independent of weight or age suggesting loss of protective effect with progression to diabetes [36].

An ambivalent role has been described for testosterone. High testosterone levels in women are associated with insulin resistance, hyperglycemia, and central obesity as well as hypertension [2, 35••]. Paradoxically, low testosterone levels in men seem to have similar effects on CVD risk factors demonstrating the duality of the mechanism of this hormone [8••, 37••]. These considerations are supported by studies providing evidence of adverse cardiometabolic consequences in women with PCOS, a state of relative testosterone excess in women, such as atherogenic lipid profiles, inflammation, fatty liver disease, hypertension, diabetes, and coagulopathy [38]. Indeed, in women with obesity, sex hormone-binding globulin (SHBG) levels are lower—associated with hyperinsulinemia and increased liver fat—a state related with higher diabetes risk in women—and further contributing to higher levels of free testosterone [8••]. Higher insulin levels resulting from higher insulin resistance contribute to an increase in insulin-mediated ovarian testosterone production in thecal cells, further contributing to sex hormone imbalance [39, 40].

## Modifiable CVD Risk Factors: Evidence for the Need for Additional and More Intensive Risk Reduction Strategies Among Women

Evidence for the need for additional and more intensive risk reduction strategies among women can be found in the study of the Prospective Studies Collaboration and Asia Pacific Studies Collaboration, where excess mortality risk among women with diabetes was investigated within other modifiable cardiovascular risk factors as BMI, total cholesterol, and systolic blood pressure compared with men [22]. These risk factors lead to higher mortality risk in women than in men. In a secondary analysis of the ADVANCE (Action in Diabetes and Vascular Disease: Preterax and Diamicron modified release Controlled Evaluation) trial, smoking contributed to an increase in CVD mortality in both sexes to a comparable extent with the exception of some evidence of a greater risk in women for major coronary events [41•]. Smoking cessation reduced all-cause mortality risk in both sexes by 30%. Several studies have indicated that not only might there be more harm at the same level of risk factors, but that women with diabetes receive insufficient medical care, leading to a worse CVD risk factor and management profile [30•, 31•, 33••, 42, 43]. This would further contribute to CVD outcome differences. Several such sex differences in CVD risk factors have been reported [8••, 33••, 42, 43].

Sex differences in CVD risk factors reported have been mostly attributed to changes after menopause, including a higher risk of hypertension in women with diabetes, higher obesity prevalence aggravating diabetes, and contributing to additional risk for CVD through inflammatory processes, ectopic lipid accumulation, insulin resistance, and hypertension [33••]. In women with type 2 diabetes, a more adverse cardiovascular risk profile has been described with higher metabolic syndrome prevalence, worse blood pressure control, and higher HbA1c. In women, hypertension, low physical activity, and high alcohol intake have been identified to be stronger predictors for acute myocardial infarction compared with men [44•]. A recently published meta-analysis of CVOTs found higher systolic blood pressure (mean difference 1.66 mmHg; 95% CI 0.90, 2.41), LDL-C (0.34 mmol/L; 0.29, 0.39), and HbA1c (0.11%; 0.09, 0.14; [1.2 mmol/mol 1.0, 1.5]) in the baseline characteristics of women compared with men participating in these trials [13••]. A register study with more than 120,000 patients with T2DM found that those females aged ≥ 60 years, when compared with males, were more obese and more likely to have higher blood pressure and dyslipidemia rates but lower rates of lipid-lowering medication, worse glycemic control, and a higher risk of retinopathy [45]. In a further cross-sectional study investigating men and women with insulin-treated T2DM aged > 60 years, women were reported to have higher levels of LDL-C and HDL-C, and higher systolic and diastolic blood pressures [46]. Furthermore, treatment goals for hypertension were achieved less often by women, despite comparable glycemia in both sexes. In a study investigating sex and gender aspects of cardiometabolic risk in T1DM, similar glycemic control but higher cholesterol in women, and higher triglyceride in men were reported [47]. Differences in cardiometabolic and CVD risk factors are already present in prediabetes and continue into diabetes: these sex differences might start early in life with higher female subclinical inflammation and coagulopathy evident from early adulthood [48]. More recent studies found that the highest relative risk sex difference for CVD events is before 60 years and highest in women with T2DM at ages 50–60 years [24•]. Differences in lipid storage and body composition driven by hormonal action seem to play an important role in this puzzle. Abdominal adiposity does increase in women with aging and a stronger obesity-diabetes risk association in women has been reported [8••]. Usually, healthy women feature higher peripheral insulin sensitivity, which is reflected in lower absolute T2DM prevalence compared with men [8••]. This means that in order to progress to T2DM, healthy women need to gain more weight than men. Indeed, women do have significantly higher BMI starting at younger ages when progressing to T2DM, particularly at younger ages [45]. Moreover, compared with men, women have a more distinct association between abdominal visceral adipose tissue and insulin resistance measured by HOMA IR and insulin secretion [49]. This has been corroborated by a further study demonstrating a higher risk for acute myocardial infarction in women with higher waist circumferences and waist-to-hip ratio compared with men [50•]. In comparison with nulliparous women, BMI was significantly higher in women with two or more children (0.6 kg/m^2^ per child) and significant worse cardiometabolic parameters with significantly lower HDL cholesterol were observed [51]. Postpartum weight changes were found to be relevant for cardiometabolic parameters with significantly higher blood pressure, HOMA-IR, LDL cholesterol, and apoB in those women who do not lose weight between 3 and 12 months after delivery [52].

## Sex Differences in Medication Effects, Side Effects, CV Risk Reduction Medications, and Adherence Among People with Type 2 Diabetes

Among participants in the FIELD study, fenofibrate was associated with greater lipid-lowering effects in women and demonstrated a 30% reduction of CVD events in women and 13% in men [53]. Rosuvastatin was associated with greater atheroma volume regression (in percent) in women, but a comparable degree of total atheroma volume regression in both sexes after 24 months. Atorvastatin has been shown to have a similar degree of atheroma regression in men and women [30•]. A higher rate of side effects for statins was observed in women with increased liver enzymes and myalgia [54]. In male and female patients with type 2 diabetes, the benefits of the use of aspirin in secondary prevention of cardiovascular events were reported [30•]. In a recent meta-analysis with 12 RCTs including > 34,000 individuals, aspirin use was found to be effective in primary prevention for MACE reduction of 11% (RR 0.89 (0.83–0.95)) with no sex interactions found [55]. During pregnancy, aspirin is recommended for preeclampsia prevention for high-risk women which includes T1DM and T2DM [56] but whether this intervention reduces later, CVD or CVD risk factors in women with diabetes is unknown.

Lower medication adherence in women than men has been reported. It has been postulated that this may be due to lower self-care activities in women as a result of higher depression rates, less problem-oriented actions, and fewer problem solving approaches which have been reported in several studies [30•]. Furthermore, higher rates of side effects leading to discontinuation of therapy were reported. A recently published meta analysis of CVOTs reported less use of beta-blockers (RR 0.93; 95% CI 0.88, 0.97), aspirin (0.82; 0.71, 0.95), and statins (0.90; 0.86, 0.93) in women compared with men but comparable use of RAAS blockers (RR 1.00; 95% CI 0.99, 1.05) [13••]. Next to the reasons above, health care providers might underestimate CVD risk in women with diabetes, which results in less prescribing of CVD protective medication and thus suboptimal CVD risk factor management.

## Sex Differences in CVD Management Among People with Diabetes

In women with T1DM, adherence to pharmacological intervention and cardio-protective measures was lower in women with guideline recommendations significantly less likely to be achieved by women for the target LDL-C levels, blood pressure, or aspirin prescription [47]. Intensive lifestyle interventions appear to be equally effective in men and women with diabetes in reducing weight, improving fitness, and maintaining healthy functioning. Similarly, prevention of progression from impaired glucose tolerance to diabetes is equally effective in men and women with a 37% lower progression rate to T2DM reported in a systematic review including 12 RCTs [57•]. Meanwhile, the DaQing study in China reported lower CVD mortality in female than male participants with prediabetes in the 23-year follow-up after lifestyle intervention [58]. These results demonstrate the effectiveness of intensive lifestyle modification in women with prediabetes and suggest that these approaches might also help to reduce CVD mortality in women with diabetes. However, this strategy needs to be verified in other cohorts. Sociocultural aspects cannot be translated easily to other cohorts and there was a substantial difference in smoking behavior—a serious CVD risk factor—between men and women within the DaQing study.

## Other Psychological and Socioeconomic Factors that Might Contribute to Increased CVD Risk Among Women

Worse metabolic control among women with diabetes is associated with lower quality of life, higher external dependency, insomnia, reduced sexual desire, and lower satisfaction with social support [30•]. Similarly higher diabetes risk among women has been associated with socioeconomic status including low education levels, low socioeconomic status in childhood, and thereafter, high job strain with low decision latitude, shift work with night shifts causing sleep deprivation [8••]. In an Austrian health survey including > 13,500 participants, only women showed an inverse association between educational level and diabetes and hypertension, with the highest risk at the lowest educational level [59]. In both sexes, overweight/obesity was higher with lower educational levels. A higher risk of T2DM was shown in Sweden with increasing age and among those of lower socioeconomic status which was accentuated in migrant women [60].

In a meta-analysis of sleep duration in the general population including > 3,500,000 participants globally, a U-shaped risk pattern was found for (i) all-cause mortality (RR 1.06 (95% CI, 1.04–1.07) per 1-h reduction and RR 1.13 (95% CI, 1.11–1.15) per 1-h increment) and (ii) for cardiovascular mortality (RR 1.06 (95% CI, 1.03–1.08) per 1-h reduction and 1.12 (95% CI, 1.08–1.16) per 1-h increment). There were no sex differences and the lowest risk at around a sleep duration of 7 h a day [61]. Again, in the general population, the Nurses’ Health Study revealed that a sleep duration of less than 5 h was closely linked with a higher incidence of hypertension in younger women [62•]. Findings of a large study including more than 700,000 people investigating the relationship of sleep duration and hypertension across age and sex found that both sexes are prone to a higher risk of hypertension with short and long sleeping durations as age increases [63]. The associations between short sleep duration were stronger among younger adults and women.

## Women with Gestational Diabetes: a Special Female Population with High Risk

Women with prior GDM are among those with the highest risk for progression to T2DM and the metabolic syndrome [31•]. Recently, a Canadian systematic review with pooled analysis including more than 5,390,000 women and a total of 101,000 cardiovascular events reported an increased risk for CVD (RR 1.98, 95% CI 1.57–2.50) up to 10 years postpartum for women with prior GDM and a subsequent diagnosis of T2DM [14••]. Interestingly, this risk was also increased in women with a history of GDM but no progression to T2DM with an RR of 1.56 (1.04–2.32). Women with GDM often suffer from hypertensive disorders in pregnancy. A twofold higher risk for CVD compared with women with normotensive pregnancies was reported in women with hypertensive disorders [64]. Both GDM and hypertensive disorders in pregnancy might add up to further increased CVD risk in later life. Intensive lifestyle intervention as reported in the “Gestational Diabetes’ Effects on Moms “(GEM) study was able to increase vigorous physical activity and decrease weight gain retention by − 0.64 kg (95% CI − 1.13, − 0.14) at 6 months postpartum in women with a history of GDM compared with usual care [65]. Women with PCOS are also at an increased risk for the development of both T2DM and CVD risk factors with their higher rates of overweight/obesity including central obesity and dyslipidemia. Although predicted, a higher risk for CVD mortality has not so far been reported due to the paucity of population-based long-term studies [66, 67].

## What Can Be Done to Reduce CVD Mortality Rates Among Women?

It may be possible to reduce CVD mortality rates among women through a range of strategies (Fig. 1). Firstly, increased awareness of the issue, including sex- and gender-specific guidelines, may be a starting point, and are consistent with the move to more personalized care. Such actions have already commenced in the treatment of those with prevalent and incident CVD [68]. This has decreased sex disparities although they are still existing and keep the “Yentl syndrome” alive (the Yentl Syndrome is the different course of action that myocardial infarctions usually follow for women than for men) [69••, 70]. Further work includes further implementation of standardized protocols, the use of m- and e-health tools to increase awareness, and the use of artificial intelligence technology.

Improved management of CVD risk factors among women is also required, again through awareness, education, guidelines, and protocols, and also possibly through earlier management of hypertension, dyslipidemia, excess weight, and hyperglycemia. Whether metabolic targets need to be lower for women is unclear [71].

Further work is needed to address gender differences in adherence which also appear to be central in the progression to T2DM and CVD. For example, depression is more prevalent in women with prediabetes and diabetes and might explain some of the differences in treatment adherence [48]. Depression can be aggravated with worse glycemic control, potentially further contributing to reduced self-management and thus increasing mortality risk. Improved depression management in women with diabetes and prediabetes may therefore address some of the CVD protection loss.

Evidence for additional and more intensive (some would say aggressive) treatment to improve CVD outcomes still needs scientific proof, but several strategies have been shown to be effective for reducing CVD events before diabetes has developed including lifestyle interventions in women with prediabetes, with a history of GDM, hypertensive disorders in pregnancy, and PCOS [57•, 72, 73••]. Further evidence is needed to open further opportunities for the prevention of CVD events among women at risk of diabetes.

Further research into sex and gender differences is important to understand the biological basis of these sex disparities further, how to improve clinical, more personalized care, alongside studies on how to increase awareness and understanding of women, their partners, and health care professionals.

The associations of sex hormones and glucose metabolism as well as other hormones such as GLP1 need further investigation, as they seem to have an important role in energy regulation and are involved in beta cell protection [36]. New findings and further research might pave the way for sex-specific targeted therapeutic modalities, as for example selective estrogen receptor modulators in postmenopausal women, GLP-1–estrogen conjugate pathways or selective ERα activation [36].

## Conclusion

We conclude that women need additional and more intensive cardiovascular risk reduction strategies beyond those currently available to men with diabetes. Women with diabetes have a higher relative CVD mortality risk, due to the loss of their “natural” protection with progression to diabetes. Evidence from recent studies demonstrates that this is especially true for younger age groups where sex disparities are more prevalent and for women with prior GDM. Whether more aggressive strategies are required such as lower metabolic targets, greater polypharmacy earlier in the disease course is unknown and warrant urgent trials.

Interventions have commenced [60], including adaptations to guidelines and protocols, but further work is needed to address gender differences in adherence through improved management of co-morbidities, e.g., depression and improved CVD risk factor management. A key area to be addressed urgently is the follow-up of women with prior GDM or hypertensive disorders in pregnancy to systematically reduce progression to T2DM and CVD through evidence-based programs and services.
